# Remarkable response to dacomitinib in a patient with leptomeningeal carcinomatosis due to *EGFR*‐mutant non‐small cell lung cancer

**DOI:** 10.1111/1759-7714.13712

**Published:** 2020-10-28

**Authors:** Shun Mizusaki, Kohei Otsubo, Toshifumi Ninomiya, Hidenobu Arimura, Yuko Tsuchiya‐Kawano, Koji Inoue

**Affiliations:** ^1^ Department of Respiratory Medicine Kitakyushu Municipal Medical Center Fukuoka Japan

**Keywords:** Dacomitinib, EGFR mutation, leptomeningeal carcinomatosis, non‐small cell lung cancer

## Abstract

Dacomitinib, a second‐generation epidermal growth factor receptor (EGFR)‐tyrosine kinase inhibitor, is a standard therapeutic option for patients with *EGFR*‐mutant non‐small cell lung cancer (NSCLC). However, its efficacy in patients with central nervous system lesions is unclear. Here, we describe a case of *EGFR*‐mutant NSCLC whose neurological symptoms were due to leptomeningeal carcinomatosis that was successfully treated with dacomitinib. After initiation of dacomitinib, the neurological symptoms of the patient were remarkably improved and leptomeningeal dissemination and brain metastases were shown to have regressed on magnetic resonance imaging (MRI) scan. To our knowledge, this is the first report showing the efficacy of dacomitinib in a patient with leptomeningeal carcinomatosis due to *EGFR*‐mutant NSCLC. The current case suggests that dacomitinib is a novel treatment option for patients with *EGFR*‐mutant NSCLC accompanied by central nervous system lesions, even those with symptomatic leptomeningeal carcinomatosis.

**Key points:**

**Significant findings of the study:**

This is the first report showing the efficacy of dacomitinib in a patient with leptomeningeal carcinomatosis due to *EGFR*‐mutant NSCLC.

**What this study adds:**

The current case suggests that dacomitinib is a novel treatment option for patients with *EGFR*‐mutant NSCLC accompanied by CNS lesions, even in those with symptomatic leptomeningeal carcinomatosis.

## Graphical abstract

Although dacomitinib is a standard therapeutic option for patients with *EGFR*‐mutant non‐small cell lung cancer (NSCLC), its efficacy in patients with CNS lesions is unclear. Here, we describe a case of *EGFR*‐mutant NSCLC with leptomeningeal carcinomatosis that was successfully treated with dacomitinib.

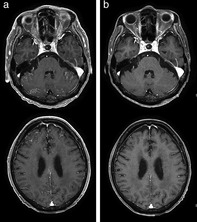

## Introduction

Dacomitinib, a second‐generation epidermal growth factor receptor (EGFR)‐tyrosine kinase inhibitor (TKI), irreversibly inhibits all members of the *ErbB* (*EGFR*, *ERBB2*, *ERBB3*, and *ERBB4*) tyrosine kinase family.^1^ Dacomitinib has been shown to prolong progression‐free survival (PFS) compared with gefitinib in patients with *EGFR* mutations^2^; however, its efficacy in patients with central nervous system (CNS) lesions is unclear. We herein present a patient with symptomatic leptomeningeal carcinomatosis due to *EGFR*‐mutant non‐small cell lung cancer (NSCLC) who exhibited a marked response to dacomitinib.

## Case report

**Figure 1:**
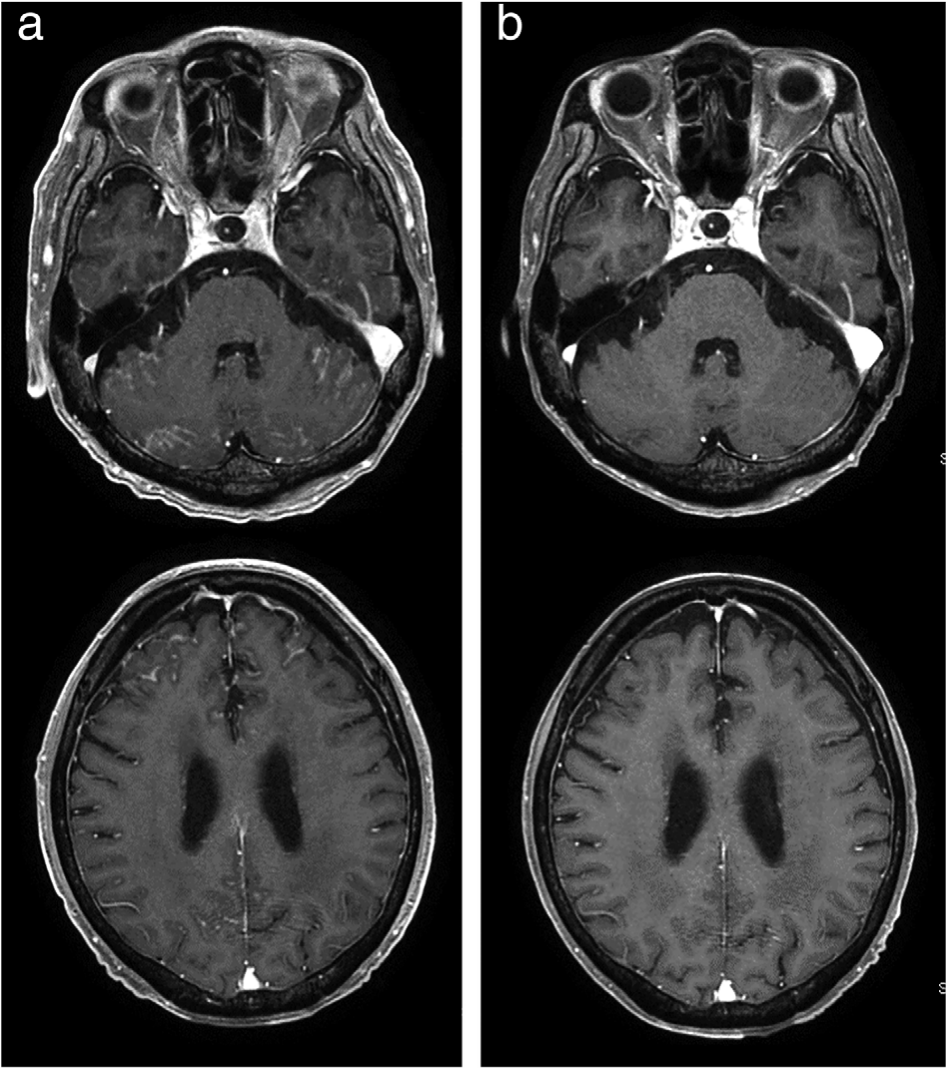
Brain magnetic resonance imaging showed (a) enhancement of the leptomeninges and small nodules of the cerebellar folia and frontal lobe, all of which diminished following treatment with dacomitinib (**b**).

Erlotinib therapy was initiated in a 72‐year‐old male patient diagnosed with stage IV lung adenocarcinoma (cT2bN3M1c) with *EGFR* mutation (exon 19 deletion) and multiple brain metastases following whole‐brain radiotherapy. However, treatment was discontinued two weeks later due to hepatotoxicity and the patient was subsequently treated with afatinib for 15 months until disease progression. Recurrence of CNS lesion was not detected, and repeat biopsy performed at the time indicated the absence of *EGFR* T790M mutation, which conferred first‐ or second‐generation EGFR‐TKI resistance.^3^ Thereafter, he received four cycles of carboplatin and pemetrexed, followed by two cycles of pemetrexed maintenance therapy until disease progression. After four cycles of docetaxel, he developed symptoms including altered consciousness, gait disturbance, and anorexia, and magnetic resonance imaging (MRI) revealed progression of leptomeningeal dissemination and brain metastases (Fig 1a). Cytological analysis of cerebrospinal fluid indicated the presence of adenocarcinoma cells, although these did not harbor the T790M mutation. Subsequently, 30 mg/day dacomitinib was initiated, which led to a remarkable symptomatic improvement, facilitating the patient's discharge in three weeks after the initiation of dacomitinib. Nine weeks after the initiation of dacomitinib, the patient showed no symptoms, leptomeningeal dissemination and brain metastases had regressed on MRI (Fig 1b), and the lung lesions continued to decrease in size.

## Discussion

Symptomatic leptomeningeal carcinomatosis is associated with a poor prognosis due to the insufficient penetration of anticancer agents into the cerebrospinal fluid, which increases the risk of treatment failure. Although some reports have suggested the efficacy of other EGFR‐TKIs for CNS lesions,^4^ the efficacy of dacomitinib for CNS lesions is unclear. A phase III study reported the superiority of dacomitinib to gefitinib as first‐line treatment of patients with *EGFR‐*mutant NSCLC based on PFS; however, patients with CNS lesions were excluded from the study. A preclinical study in mice suggested the capability of dacomitinib to cross the blood‐brain barrier (BBB),^5^ and a phase II study of glioblastoma in humans demonstrated high levels of dacomitinib concentration in intracranial tumor tissues.^6^ Pharmacokinetically, dacomitinib is not a substrate of P‐glycoprotein (P‐gp/ABCB1) or breast cancer resistance protein (BCRP/ABCG2) that transport their substrates, such as gefitinib and erlotinib, back to the blood at the BBB.^7^, ^8^, ^9^ Although other EGFR‐TKI rechallenge has been reported to be effective in some cases,^10^ we found that dacomitinib efficiently penetrates the BBB, demonstrating the potential to be a better therapeutic option for leptomeningeal metastases of *EGFR*‐mutant NSCLC than other EGFR‐TKIs. Furthermore, it is possible that dacomitinib is beneficial for patients with CNS lesions who have a history of treatment with other EGFR‐TKIs, as illustrated in the present case.

To our knowledge, this is the first report showing the efficacy of dacomitinib in a patient with leptomeningeal carcinomatosis due to *EGFR*‐mutant NSCLC. The current case suggests that dacomitinib is a novel treatment option for patients *EGFR*‐mutant NSCLC accompanied with CNS lesions, even in those patients with symptomatic leptomeningeal carcinomatosis.

## Disclosure

The authors declare no conflicts of interest associated with this manuscript.
